# *Clostridioides difficile* in feral horse populations in Australia

**DOI:** 10.1128/aem.02114-24

**Published:** 2025-04-02

**Authors:** Natasza M. R. Hain-Saunders, Daniel R. Knight, Andrea Harvey, Mieghan Bruce, Brian A. Hampson, Thomas V. Riley

**Affiliations:** 1 Biosecurity and One Health Research Centre, Harry Butler Institute, Murdoch University 625323 , Murdoch, Western Australia, Australia; 2 Department of Microbiology, PathWest Laboratory Medicine, Queen Elizabeth II Medical Centre 503908 , Nedlands, Western Australia, Australia; 3 School of Biomedical Sciences, The University of Western Australia, Queen Elizabeth II Medical Centre 550238 , Nedlands, Western Australia, Australia; 4 Centre for Compassionate Conservation, School of Life Sciences, Faculty of Science, University of Technology Sydney 170529 , Broadway, New South Wales, Australia; 5 School of Veterinary Medicine, Murdoch University 172103 , Murdoch, Western Australia, Australia; 6 Independent Researcher, Brisbane, Australia; 7 School of Medical and Health Sciences, Edith Cowan University 204605 , Joondalup, Western Australia, Australia; Universidad de los Andes, Bogotá, Colombia

**Keywords:** *Clostridioides difficile*, *C. difficile*, horse, One Health

## Abstract

*Clostridioides difficile* is a known cause of diarrhea and colitis in human and non-human animals. While *C. difficile* is regularly isolated from domesticated horses, little is known about its prevalence in wild or feral populations. In Australia, the horse population encompasses a mix of both domesticated and feral animals, with the feral population of 400,000 estimated to be the largest in the world. This study investigated the presence and characteristics of *C. difficile* in Australian feral horses and evaluated their potential as a source or reservoir of *C. difficile* in the wider community. Fecal samples (*n* = 380) were collected from free-roaming feral horses from five Australian jurisdictions and cultured for *C. difficile*. Isolates were characterized by PCR ribotyping and toxin profiling. Antimicrobial susceptibility testing was performed for fidaxomicin, vancomycin, metronidazole, rifaximin, clindamycin, erythromycin, amoxicillin-clavulanate, moxifloxacin, meropenem, and tetracycline. *C. difficile* was isolated from 45 of the 380 samples (11.8%)—one-third of that seen in recent studies on Australian domesticated horses but consistent with wild animal species worldwide. Forty ribotypes (RTs) were identified, 28 of which (70%) were novel; other RTs had been previously reported in humans, livestock, and soils. Eighteen toxigenic *C. difficile* strains were isolated, of which eight contain binary toxin genes. Strains were largely susceptible to the antimicrobial agents tested. This investigation provides preliminary information on *C. difficile* in feral horses in Australia and allows a comparison with their domestic counterparts. The findings support the hypothesis that all horse feces represent a potential source of *C. difficile* in the community.

**IMPORTANCE:**

*Clostridioides difficile* poses an ongoing threat to healthcare in the community, with increasing evidence of transmission outside the hospital setting. In keeping with a One Health model of dispersion, investigations into this microorganism within the wider environment are vital to understanding this evolving epidemiology. Australia has the biggest population of feral horses in the world, and this study of *C. difficile* in feral horses provides insight into the role of non-domesticated animals in the dissemination of *C. difficile*. Examination of prevalence and characterization of isolates provides a baseline for evaluating the effect of antimicrobials and other factors associated with domestication on equine *C. difficile* infection.

## INTRODUCTION

*Clostridioides* (*Clostridium*) *difficile* is a gram-positive anaerobic bacillus that causes diarrhea and colitis in human and non-human animals. As a spore-forming bacterium, the ability of *C. difficile* to persist for long periods in the environment and resist common bactericidal agents, such as alcohol, creates unique challenges for disease prevention and control (1). Furthermore, increasing antimicrobial resistance (AMR), an epidemiological shift in human disease toward infection in the community, and mounting evidence of zoonotic links further highlight the need for more rigorous investigation beyond traditional sources (2–5).

In horses, *C. difficile* is a known cause of toxin-mediated gastrointestinal disease, impacting both animal welfare and the financial integrity of equine industries. Transmission is by the fecal–oral route with impact ranging from asymptomatic carriage to diarrhea, necrotizing enteritis, and associated complications resulting in death (6). As hindgut fermenters, horses are sensitive to disruption of gut microflora and can show a rapid decline when infected (7). Mortality rates of confirmed equine *C. difficile* infection (CDI) cases range between 26% and 80% (8–10). In Australia, recent studies reported the presence of *C. difficile* in 31–38% of domestic horses, with genetic analysis also identifying clonal strains between horse and human populations (11). Despite this, investigations into feral and non-domestic equine populations are lacking.

Australia has the largest population of feral horses (often called brumbies) in the world, comprising an estimated 400,000 feral horses, with origins dating back to the early 1800s (12, 13). Distribution extends to all Australian mainland jurisdictions, encompassing a variety of habitats from semi-arid plains through to tropical grasslands and sub-alpine forests (14). Home ranges for feral horse social groups span between 30 and 70 km^2^, making the potential for dissemination of infectious agents significant and far-reaching (15). Much of these areas extend through Australia’s vast open pastoral areas, creating further dispersal risk at the wildlife–livestock–human interface through shared water sources and environment.

Investigations into *C. difficile* in wild and feral animals can help to establish a broader understanding of disease epidemiology as well as provide insights into the evolution and ecology of the bacterial species (16). Remote animal populations also provide a basis for comparison as subjects with limited exposure to suspected risk factors such as antimicrobials and the hospital environment. It should be noted here that the terms “wild” and “feral” in relation to animals can be variably interpreted with no international standards for terminology. For the purposes of this study, “wild” is defined as a non-domesticated animal, which is indigenous to the region, and the term “feral” as a free-roaming, self-sustaining, non-indigenous animal (whether it be an individual transitioned from domestication or one of its descendants).

This study aimed to investigate the epidemiology of *C. difficile* in Australian feral horse populations, to identify points of comparison with their domestic counterparts, and to evaluate their potential as a source or reservoir for disease dissemination.

## RESULTS

### Epidemiology

Overall, *C. difficile* was isolated from 45 of the 380 feral horse fecal samples (11.8%, 95% CI: 9.0–15.5%). Recovery at individual sampling sites, however, varied considerably (range, 0–33.3%). The proportion of samples with *C. difficile* in the Northern Territory (3.0%) was lower than Western Australia (16.4%; *P* = 0.002) and New South Wales/Victoria (15.1%; *P* = 0.002) with no difference between other jurisdictions. Table 1 summarizes the proportions of *C. difficile* isolated from Australian feral horses for the collection sites and jurisdictions.

**TABLE 1:** *C. difficile* proportion positive isolated from feral horse feces by location

Sample origin	Samples (*n*)	*C. difficile*-positive samples	Proportion positive (%)	95% CI
Western Australia	**73**	**12**	**16.4%**	(**9.7%; 26.6**)
Ashburton	24	2	8.3%	(2.3%; 25.9%)
Meekatharra	9	0	0.0%	(0.0%; 29.9%)
Lake Logue	18	6	33.3%	(16.3%; 56.2%)
Cape Arid	10	2	20.0%	(5.7%; 50.9%)
Merivale	12	2	16.7%	(4.7%; 44.8%)
Queensland	**21**	**2**	**9.5%**	(**2.6%; 28.9%**)
Northern Territory	**101**	**3**	**3.0%**	(**1.0%; 8.4%**)
New South Wales/Victoria	**185**	**28**	**15.1%**	(**10.7%; 21.0%**)
Blue Mountains	25	6	24.0%	(11.5%; 43.4%)
Bogong High Plains	7	0	0.0%	(0.0%; 35.4%)
Cooleman	73	14	19.2%	(11.8%; 29.6%)
Cowombat	54	4	7.4%	(2.9%; 17.5%)
South Kosciuszko N.P.	26	4	15.4%	(6.1%; 33.5%)
Total	**380**	**45**	**11.8%**	(**8.9%; 15.7%**)

**Fig 1:**
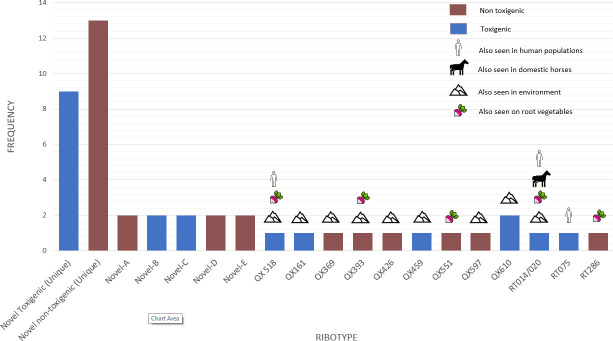
Characteristics of *C. difficile* isolated from feces of 45 Australian feral horses. Internationally recognized strains prefixed “RT” (*n* = 3) and locally identified strains prefixed “QX” (*n* = 10). Toxigenic strains contain at least one of the toxin genes *tcdA* (A+), *tcdB* (B+), or *cdtA/B* (CDT+). Novel strain data points titled “Unique” comprise the total number of novel strains in this category, which were only isolated once in this study (*n* = 22). Novel strain data points that are numbered (A, B, C…) indicate individual novel strains with a frequency of more than one (*n* = 10).

In total, 40 different *C. difficile* ribotypes (RTs) were identified across all horse samples. This comprised a combination of novel (*n* = 28) and previously described (*n* = 12) RTs, including RTs 014/020 and RT 075, which have been reported in human CDI. Some of the other RTs had been reported also in Australian root vegetables, soils, and water. Figure 1 shows the RT frequencies of all *C. difficile* strains isolated and an indication of other sources of similar strains.

**Fig 2:**
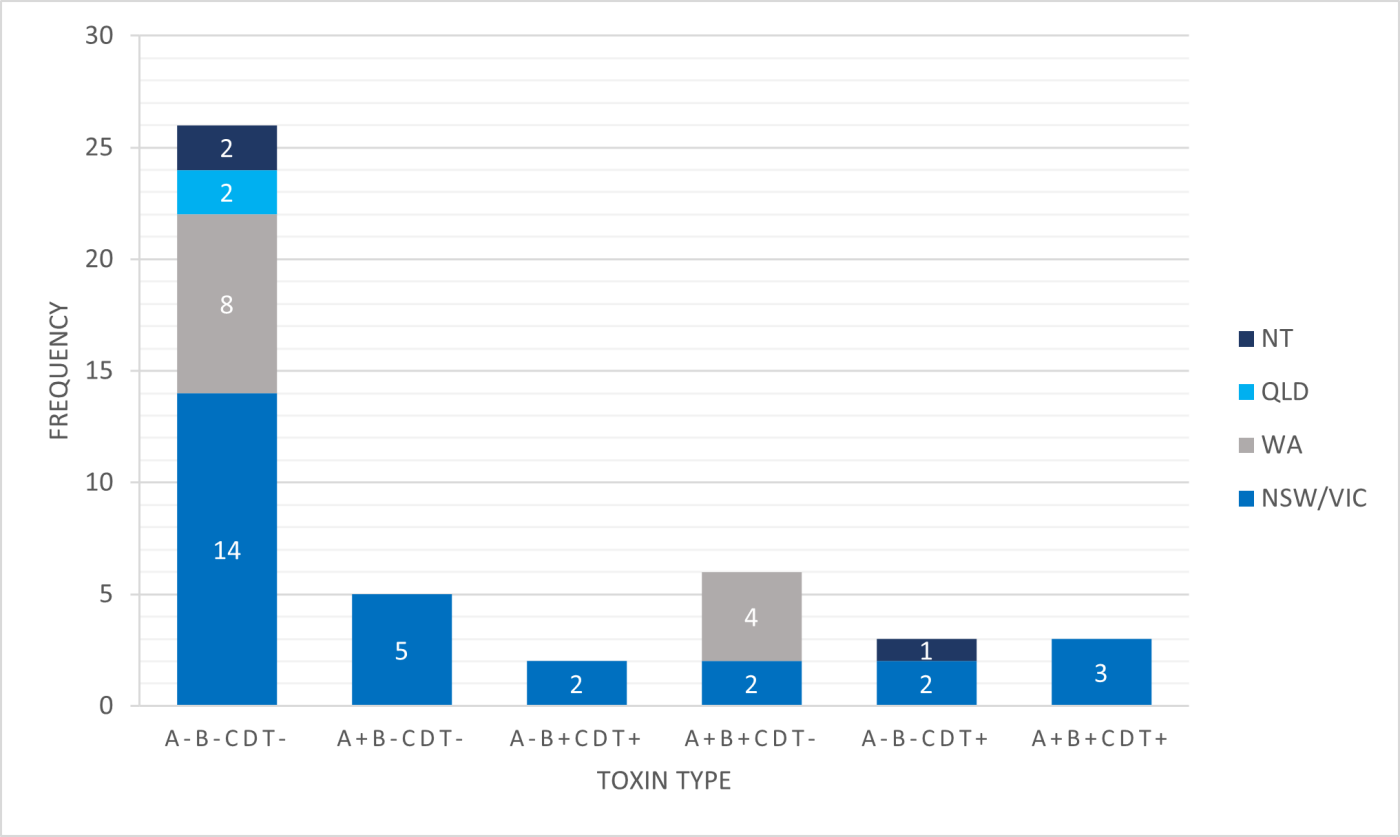
Toxin gene profiles of *C. difficile* strains isolated from Australian feral horse feces. Toxigenic strains are those which contain at least one of the toxin genes *tcdA* (A+), *tcdB* (B+), or *cdtA/B* (CDT+).

There were six different toxin gene profiles identified, with frequencies of each outlined in Fig. 2. Forty-two percent of *C. difficile* isolates (*n* = 19) were toxigenic, containing at least one of the toxin genes *tcdA* (A+), *tcdB* (B+), or *cdtA/B* (CDT+). Of these, six strains contained binary toxin genes (five in novel RTs, plus one in RT075), three of which contained binary toxin genes only. In addition, five strains contained the toxin A gene only.

### Antimicrobial susceptibility testing (AST)

**Fig 3:**
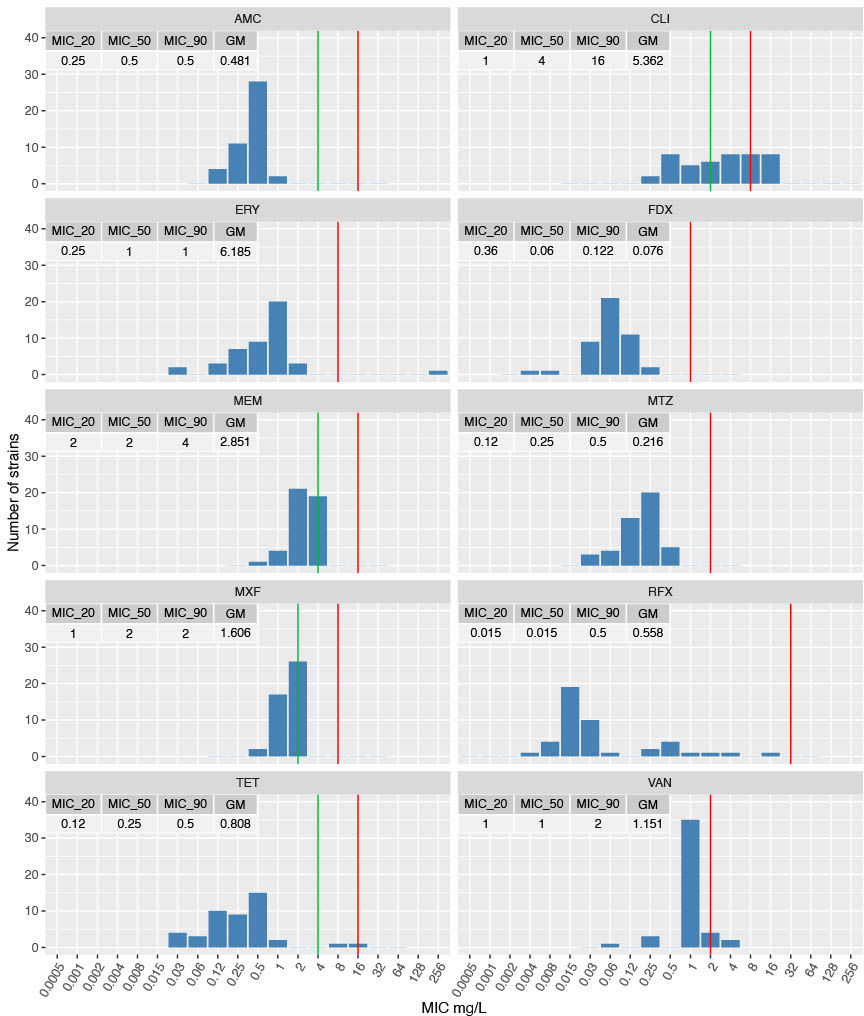
MIC distributions for 10 antimicrobial agents against 45 Australian feral horse strains of *C. difficile*. Breakpoints are indicated with green (susceptible) and red (resistant) vertical lines. AMC, amoxicillin/clavulanate; CLI, clindamycin; ERY, erythromycin; FDX, fidaxomicin; MEM, meropenem; MTZ, metronidazole; MXF, moxifloxacin; RFX, rifaximin; VAN, vancomycin.

MIC distributions for the 10 antimicrobial agents tested against the *C. difficile* strains isolated from Australian feral horses are shown in Fig. 3. These strains were largely susceptible to the 10 antimicrobial agents. Resistance to four agents was identified, comprising clindamycin (*n* = 16, 36%), erythromycin (*n* = 1, 2%), tetracycline (*n* = 1, 2%), and vancomycin (*n* = 2; 4%), the latter of which is used in the treatment of human CDI. Susceptibility to fidaxomicin, also used to treat human CDI, remained high. All strains were susceptible to metronidazole (MIC <2 mg/L), the preferred agent for the treatment of CDI in animals. One Western Australian strain (LL011) displayed resistance to three (clindamycin, erythromycin, and tetracycline), deeming it multi-drug resistant.

**Fig 4:**
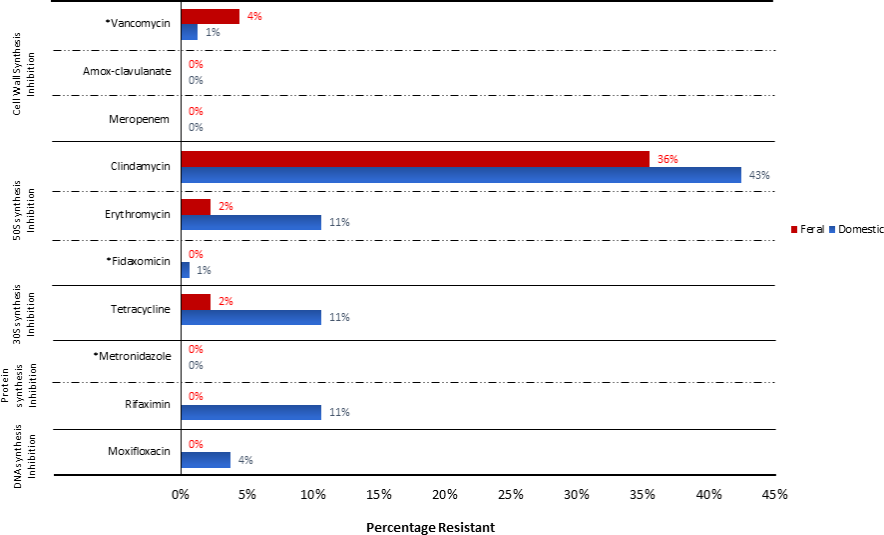
Comparison of resistance to 10 antimicrobial agents in strains of *C. difficile* isolated from Australian feral (*n* = 45) and domestic (*n* = 160) horses. *Agents currently recommended for the treatment of *C. difficile* in humans (fidaxomicin, metronidazole, and vancomycin) and horses (metronidazole only).

Figure 4 presents a comparison of the percentage of AMR in feral horse *C. difficile* strains compared to domestic horse data sets described in Hain-Saunders et al. (11). Resistance in domestic horse isolates appeared consistently higher than that in feral horses except for vancomycin. Despite this, the difference between the two populations was only statistically significant for rifaximin (*P* = 0.02).

### Atypical phenotype

**Fig 5:**
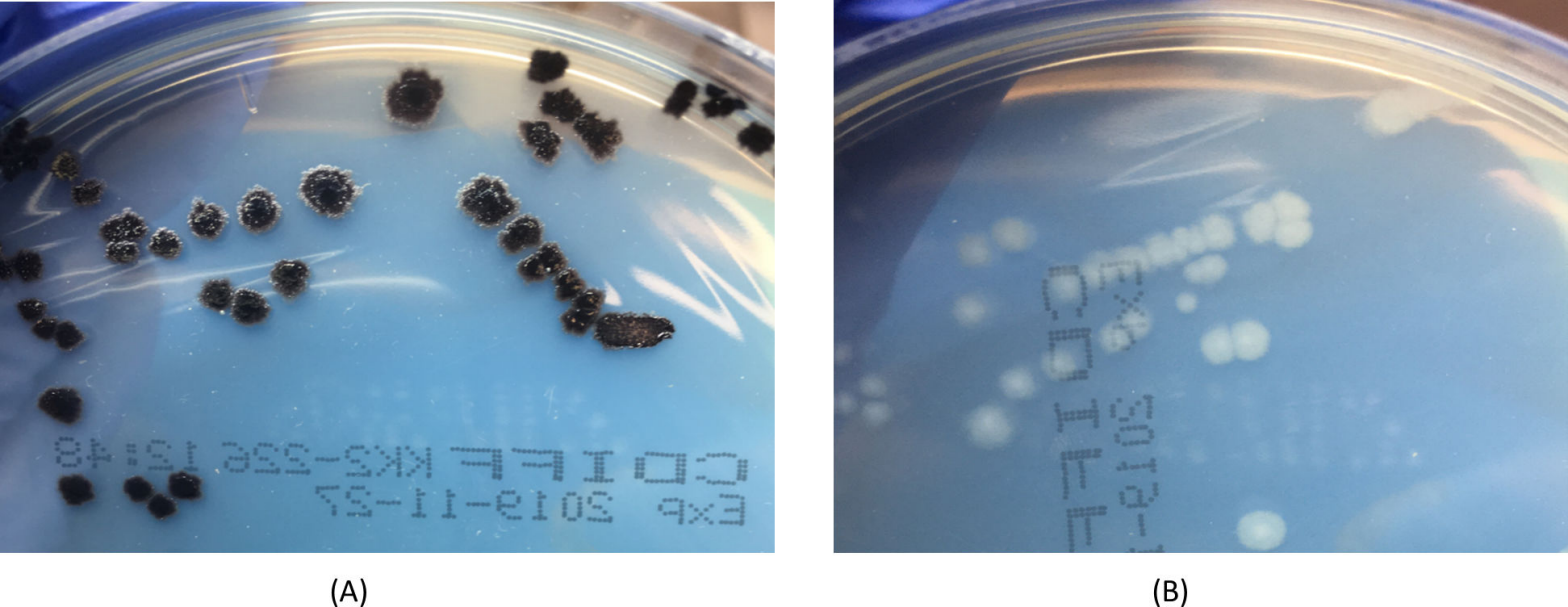
Images showing (**A**) the typical appearance of *C. difficile* growth on CHROMID *C. difficile* agar (bioMérieux) after 48 h with black colonies of control strain *C. difficile* RT078 and (**B**) the growth of esculin hydrolysis-negative *C. difficile* strain H285 (isolated in this study) after 48 h growth with white colonies on CHROMID.

One noteworthy strain isolated from feral horse feces produced white colonies on CHROMID *C. difficile* agar (bioMérieux). Typically, growth of *C. difficile* on CHROMID *C. difficile* agar gives black colonies due to esculin hydrolysis. However, despite verification as *C. difficile* by MALDI-TOF, microscopy, and phenotypic characteristics on horse blood agar, colonies of New South Wales strain H285 demonstrated a white, ground-glass appearance on CHROMID as shown in Fig. 5 compared to the typical black colonies of a control strain. This non-toxigenic strain appeared to be a novel RT.

## DISCUSSION

This study is the first known investigation into *C. difficile* in feral horse populations. The data provide valuable information on the prevalence of *C. difficile* in animals at the wildlife–livestock–human interface as well as baseline data for evaluating the effects of antimicrobials and other domestication factors on *C. difficile* prevalence and characteristics.

### *C. difficile* isolation

The overall recovery of *C. difficile* in feral horse feces (11.8%) was consistent with *C. difficile* prevalence identified in wild mammal populations such as polar bears (16.8%), wild bears (15%), and migratory bats (19.4%) (16, 17). The consistency seen in these limited subsets is suggestive of an underlying reservoir in wild and feral populations. The considerable variation in the prevalence of *C. difficile* seen between collection sites was similar to that seen in domestic horse populations and may reflect transient colonization with *C. difficile,* although site and jurisdictional biogeographical variations should be considered. Such variability has been reported previously in horse longitudinal studies and in other livestock populations and has been attributed to short-term stress factors such as feed changes, transportation, and seasonal changes, which can lead to a disruption to the natural gut flora (11, 18–20).

Investigations into *C. difficile* in horses to date have focused on companion animals and racehorses, with reported *C. difficile* carriage ranging between 0 and 14% in healthy adult horses, 5 and 63% in diarrheic horses, and 16 and 29% in foals (1, 21–25). This categorized approach to prevalence reporting reflects the risk factors associated with *C. difficile*. Age and health status are two major factors in disease prevalence and transmission. Young human and non-human animals consistently show high rates of colonization with *C. difficile*, often attributed to their developing gut microbiome (25). In the current study, the blind testing of feral horse fecal samples with limited knowledge of the host, particularly health status, created some difficulties in analysis and the extent to which these factors impacted the results. Collection of whole pellets of horse feces from the feral horses, however, indicated that these horses were not diarrheic at the time of sampling.

*C. difficile* recovery from Australian feral horse feces (12%) was significantly less than from Australian domestic horses with (38%, *P* < 0.001) and without (31%, *P* < 0.001) gastrointestinal signs using the same experimental approach and methods (11). While the higher population density of domesticated horses and proximity to other animals and humans increases the potential for dispersal, the use of antimicrobials in the domestic populations is likely to be the most important factor. It has been long established that antimicrobial use is a factor influencing CDI in both humans and animals, through the disruption of the gut microbiota (26). The role of the existing microbiota in wider disease establishment is becoming increasingly apparent (27). In the case of *C. difficile*, it is thought that commensal bacteria compete for nutrients and adhesion sites. The use of antimicrobials not only disrupts this natural barrier, but studies have also suggested that certain antimicrobials may increase adhesin and toxin gene expression, leading to increased pathogenicity (28, 29).

Antimicrobials are widely used in domesticated horse populations, one of the most common being ceftiofur (30, 31). Ceftiofur significantly disrupts commensal bacterial counts in the horse hindgut for at least 1 week after administration, with the appearance of *C. difficile* within 24 h of administration (32). Despite a national action plan calling for the urgent establishment of antimicrobial stewardship programs in veterinary practices across Australia, implementation remains limited (33, 34).

### Toxin profile variability

Overall, 42% of all strains isolated in this study were toxigenic. This appears comparatively lower than previous investigations into *C. difficile* in horses and other livestock (11, 21, 35, 36). The reasons behind this lower toxigenicity were not clear; however, the absence of known diarrheic samples in this study likely played a role.

Although symptomatic infection is related to the presence of toxin-forming strains of *C. difficile*, it has been shown experimentally that non-toxigenic strains can acquire toxin genes through horizontal gene transfer (37–39). While the frequency at which this occurs is unknown, horizontal gene transfer is thought to have been a factor in the emergence of hypervirulent strains in recent times such as RT 027 (40). Epidemiological studies of both toxigenic and non-toxigenic strains therefore remain critical for effective surveillance of emerging strains.

The isolation of toxigenic strains containing only toxin A or binary toxin genes in this study is also of great concern. Virulence investigations of such strains in hamster models have reported both severe disease and death; however, their discovery in clinical disease is relatively recent (41). The ability of these mono-toxin strains to evade certain laboratory tests needs to be considered in the use and development of *C. difficile* laboratory protocols and testing schemes (42). While two-step testing is recommended in both animal and human *C. difficile* disease diagnosis, this has yet to gain widespread adoption in equine veterinary practice (43–45). Inconsistency and a lack of standardization in diagnostic protocols have been reported as being a major hindrance to the clinical diagnosis of horse colitis cases and understanding of the equine gut microbiota (46, 47). These issues, combined with insufficient testing regimes, pose a significant and ongoing risk to both equine and human health.

### Diversity of RT patterns

Only 13 of the 45 *C*. *difficile*-positive samples’ RTs were previously identified, having been reported in lawns, vegetables, and humans (48, 49). The remaining 32 samples (71%) contained RTs which were novel to our laboratory. As investigations into community-associated CDI gain momentum and exploration into environmental sources of *C. difficile* continues, it is likely more novel RTs will be identified, particularly in remote regions. The high diversity of RTs and the minimal overlap between isolates from feral horses compared to domestic horse populations are not surprising. Given the remote locations of the feral populations, this likely indicates that regional strains may have evolved in isolation from the more common strains circulating in domesticated populations and urban settings. Although feral horses are not native to Australia, many of the populations sampled have been established within the remote regions since the early 19th century (15), allowing time for these unique strains to develop and disperse through local populations. Evidence for such localized evolution has come to light in recent genomic investigations of clade 5 ST11 strains, which proposed the coevolution of the closely related RT 078 and RT 126, occupying the same niche within different geographical regions, and possibly explaining the distinct absence of the former within Australia (4).

This may also be the case for the identification of isolates with atypical characteristics as seen with the isolation of an esculin hydrolysis negative strain. Esculin hydrolysis is a biochemical characteristic of *C. difficile* exploited in some differential agar, including CHROMID used in this current study. On these plates, the hydrolysis of esculin within the agar creates a black pigmentation and results in *C. difficile* strains appearing as black colonies (50). Isolates that do not possess this characteristic appear as white colonies on CHROMID and therefore bypass the differential function of the agar. There have been reports of esculin hydrolysis-negative *C. difficile* RTs in clinical settings (51, 52) as well as in soils (53). Although the isolate identified in the current study was non-toxigenic, this presents an expanding issue in the emergence of esculin hydrolysis-negative *C. difficile* strains and concern for false-negative results.

### Antimicrobial susceptibility testing

Antimicrobial susceptibility results were similar to studies of domestic horses in Australia, showing a high proportion of clindamycin resistance and limited resistance to erythromycin and tetracycline. Interestingly, two feral horse strains from different geographical regions were resistant to vancomycin with MICs of 4 mg/L (breakpoint >2 mg/L), an agent used in the treatment of CDI in humans. Mutation in the VanSCD sensor histidine kinase and VanRCD response regulator of the vanG operon-like gene cluster vanGCD has been proposed as the primary mechanism for vancomycin resistance in *C. difficile*, although individual causes were not explored in this study (54).

Given the limited exposure of feral animals to antimicrobial agents, however, this likely indicates the existence of reservoirs of resistance due to spontaneous mutation (as opposed to that induced by exposure) outside domestic and clinical settings and further supports the need for a more formalized means of AMR surveillance and antimicrobial use monitoring in animal populations in Australia. For an organism that the US CDC named in the top five posing an urgent AMR threat to public health, complacency can no longer be accepted (55).

### Conclusion

The diversity of strains and unique characteristics identified in this study indicate that further investigation into *C. difficile* outside of traditional settings is required. While diagnostic testing focuses on the mere presence of disease, an urgent need for molecular typing as an ongoing surveillance measure is becoming increasingly apparent. The findings in this study support the hypothesis that horses—whether feral or domestic—represent a possible source/reservoir for *C. difficile* dispersion through interactions with humans, other animals, and the environment. This highlights the need for a holistic “One Health” approach to *C. difficile* research, and prevention and control of disease.

## MATERIALS AND METHODS

### Study population and sample collection

**Fig 6:**
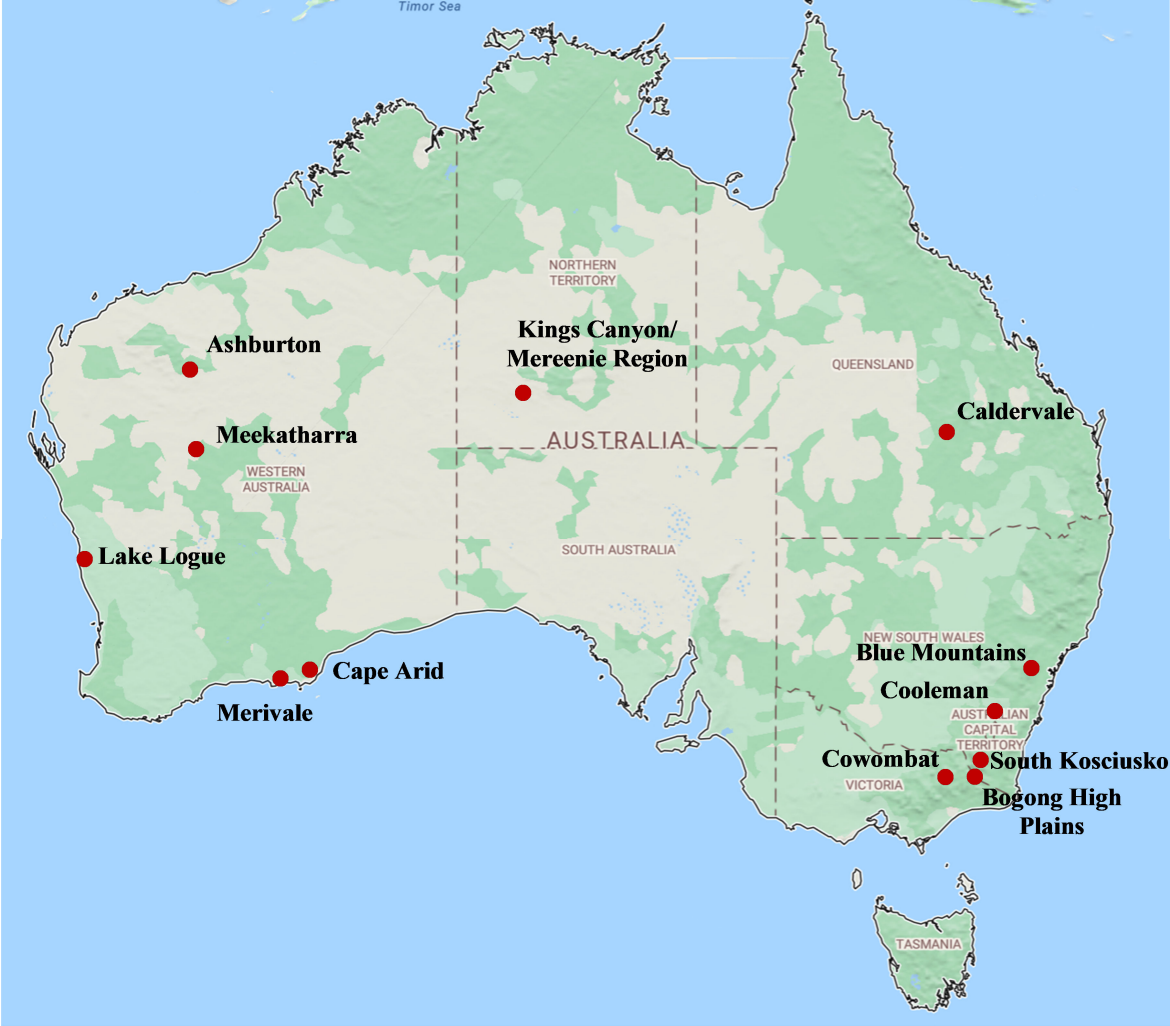
Feral horse fecal sample collection sites across Australia. Map data © 2019 GBRMPA, Google.

Fecal samples (*n* = 380) were collected between December 2016 and December 2020 from 11 locations across five Australian jurisdictions, including the States of Western Australia, Queensland, New South Wales and Victoria, and the Northern Territory (Fig. 6). Western Australian and Northern Territory collection sites were identified in collaboration with brumby associations, agricultural landowners, and traditional landowners based on known population territories and reports of recent sightings. Where feces were fully formed, whole individual fecal pellets were collected to reduce contamination of the internal fecal matter from the environment, and, where allowed, environmental samples were also taken close to where the fecal samples were collected to assess for potential contamination. In Queensland, samples were collected from feral horses during trapping and relocation programs, while New South Wales and Victorian samples were taken during a population ecology, health, and welfare survey with detailed sample collection procedures further outlined by Harvey et al. (56). New South Wales and Victorian data are combined in data comparisons reported here due to the close proximity of some sampling sites in these jurisdictions.

### Culture

Fecal samples were cultured for *C. difficile* using previously described methods (11). Briefly, individual fecal pellets were carefully cut on the transverse plane, and a portion was extracted from the core to avoid surface environmental contamination. Approximately 10 g was added to a 90 mL selective brain heart infusion broth supplemented with 5  g/L yeast extract, 1  g/L l-cysteine, 1  g/L taurocholic acid, 250  mg/L cycloserine, and 8  mg/L cefoxitin (BHIB-S) (PathWest Media, Mt Claremont, WA) and incubated with lids loose for 7 days in an A35 anaerobic chamber (Don Whitley Scientific Ltd, Yorkshire, UK) at 35°C with an atmosphere of 80% nitrogen, 10% hydrogen, 10% carbon dioxide, and a relative humidity of 75%. The cultures were then alcohol shocked at a 1:1 ratio for 1 h to select for spores. A 10 µL aliquot was streaked onto a ChromID *C. difficile* agar plate (bioMérieux, Marcy-l'Étoile, France) and incubated anaerobically for 48 h. Putative *C. difficile* colonies were sub-cultured onto horse blood agar and incubated anaerobically for a further 48 h. *C. difficile* was confirmed by characteristic ground-glass colony appearance, distinct barnyard odor, and chartreuse fluorescence under UV light (~360 nm).

### *C. difficile* characterization

Multiplex PCR and capillary electrophoresis were used as previously described for ribotyping and toxin gene characterization (57). RT banding patterns were visualized using QIAxcel ScreenGel software (Qiagen, Venlo, Netherlands). Densitometric curve and band pattern analysis was performed using the Bionumerics v7.1 software package (Applied Maths, Sint-Martens-Latem, Belgium) based on the Dice coefficient and Pearson correlation, and comparison was made with a database of over 10,000 strains of both internationally recognized and local strains. Toxin gene profiles were determined by the presence of *tcdA* (toxin A), *tcdB* (toxin B), and *cdtA* and *cdtB* (binary toxin).

### AST

The antimicrobials fidaxomicin, vancomycin, metronidazole, rifaximin, clindamycin, erythromycin, amoxicillin-clavulanate, moxifloxacin, meropenem, and tetracycline were chosen for antimicrobial testing based on their usage in veterinary practice, human healthcare, and CDI treatment and prevention. MICs of these agents were determined using the agar incorporation method described by the Clinical and Laboratory Standards Institute (CLSI) (58). Clinical breakpoints used for vancomycin and metronidazole were recommended by the European Committee on Antimicrobial Susceptibility Testing (59), while those for fidaxomicin and rifaximin were proposed by the European Medicines Agency (60) and O’Connor et al. (61). All other breakpoints were based on CLSI recommendations (62). MIC50, MIC90, and geometric mean MIC values were calculated for all antimicrobials.

### Comparative analysis

Statistical analyses were performed using the Chi-squared test, with a two-tailed *P* value < 0.05 considered significant. All comparisons to domestic horse populations utilized data sets established and described in Hain-Saunders et al. (11).
